# Cumulative childhood trauma and complex psychiatric symptoms in pregnant women and expecting men

**DOI:** 10.1186/s12884-021-04327-x

**Published:** 2022-01-04

**Authors:** Julia Garon-Bissonnette, Marie-Ève Grisé Bolduc, Roxanne Lemieux, Nicolas Berthelot

**Affiliations:** 1 Department of Psychology, Université du Québec à Trois-Rivières, Québec, Canada; 2 Centre d’études interdisciplinaires sur le développement de l’enfant et la famille, Québec, Canada; 3 CERVO Brain Research Center, Québec, Canada; 4 Interdisciplinary Research Center on Intimate Relationship Problems and Sexual Abuse, Québec, Canada; 5 Groupe de recherche et d’intervention auprès de l’enfant vulnérable et négligé, Québec, Canada; 6 Department of Nursing Sciences, Université du Québec à Trois-Rivières, Trois-Rivières, PO Box 500, Québec, Canada

**Keywords:** child abuse, complex trauma, symptom complexity, mental health, pregnancy, prenatal, antenatal, mothers, fathers

## Abstract

**Background:**

Women and men having been exposed to childhood trauma would be at high risk of various mental health symptoms while awaiting a child. This study aimed to evaluate the association between cumulative childhood trauma and the accumulation of symptoms belonging to different psychiatric problems in pregnant women and expecting men.

**Methods:**

We first examined prevalence rates of childhood trauma across our samples of 2853 pregnant women and 561 expecting men from the community. Second, we evaluated the association between cumulative childhood trauma and symptom complexity (i.e., the simultaneous presentation of symptoms belonging to multiple psychiatric problems) using subsamples of 1779 pregnant women and 118 expecting men. Participants completed self-reported measures of trauma (Childhood Trauma Questionnaire) and psychiatric symptoms (PTSD Checklist for DSM-5; Kessler Psychological Distress Scale; State-Trait Anger Expression Inventory-2; Self and Interpersonal Functioning Scale).

**Results:**

Trauma was more frequent in pregnant women than in expecting men and in participants reporting sociodemographic risk factors than in those not reporting any. A dose-response relationship was observed between the number of different traumas reported by pregnant women and expecting men and the complexity of their psychiatric symptoms, even when controlling for the variance explained by other risk factors. Women having been exposed to cumulative childhood trauma were 4.95 times more at risk of presenting comorbid psychiatric problems during pregnancy than non-exposed women.

**Conclusions:**

Childhood trauma is frequent in the general population of pregnant women and expecting men and is associated with symptom complexity during the antenatal period. These findings call for delivering and evaluating innovative trauma-informed antenatal programs to support mental health and adaptation to parenthood in adults having been exposed to childhood trauma.

**Supplementary Information:**

The online version contains supplementary material available at 10.1186/s12884-021-04327-x.

## Background

About one third of adults report having been exposed to childhood trauma under the form of abuse or neglect before 18 years old [1, 2]. Prevalence rates would differ according to sex, with women being more likely to report sexual abuse and men physical abuse [1]. The prevalence of childhood trauma in pregnant women and expecting men remains uncertain [3], but existing data suggest that childhood abuse and neglect is also frequent in this population [4, 5].

A vast body of knowledge has indicated that childhood trauma exerts an important toll on maternal mental health during the perinatal period. Indeed, women with histories of childhood trauma report higher rates and severity of depression, post-traumatic stress disorder (PTSD), dissociation, and personality disorders than non-exposed women during pregnancy [6–10] and postpartum [11, 12]. Although studies on fathers are sparse, previous research also suggests that exposure to childhood trauma would be associated with an increased severity of symptoms across multiple diagnoses among expecting men [6].

Previous research on mental health in pregnant women and expecting men mainly considered psychiatric disorders in isolation, whereas the evidence suggests that exposure to trauma could be an enhancer for the progressive accumulation of deficits and difficulties across multiple domains of functioning [13–17]. Therefore, expecting parents exposed to childhood trauma may not only display more severe symptoms than those without trauma, but may also have a complex clinical profile characterized by the simultaneous presentation of symptoms belonging to multiple psychiatric disorders [18], which previous research has referred to as the level of symptom complexity [13–15, 17, 19]. To our knowledge, only a handful of studies examined whether childhood trauma was associated with complex psychiatric symptomatology in pregnant women. First, in a community sample of 1 581 pregnant women, those having been exposed to childhood trauma were more likely to report a combination of PTSD and depression or affect dysregulation than non-exposed women [4]. More recently, Anastas and colleagues [20] reported that the accumulation of adverse childhood events was positively correlated with a score of psychopathology derived from a questionnaire assessing anxiety, depression, dissociation, PTSD and anger in 36 pregnant teens. Understanding the determinants of complex comorbid symptoms in pregnant women and expecting men is important considering that such symptoms are more difficult to treat using pharmacological [21] and psychological approaches [22], have more severe impacts on functioning [23], and could therefore be more likely to affect parenthood and offspring development than non-comorbid disorders.

The present study aims to evaluate the association between cumulative childhood trauma (defined as the experience of multiple forms of abuse and neglect before 18 years old) and the complexity of psychiatric symptoms in pregnant women and expecting men. Building on previous studies reporting a linear relationship between cumulative childhood trauma, symptom complexity and psychiatric comorbidities in a non-obstetric population [13–15, 17, 18], we hypothesized that cumulative childhood trauma would be associated with greater symptom complexity and comorbid psychiatric problems. Given the limited number of studies evaluating the prevalence of trauma in community samples of expecting parents, we began by estimating the rates and characteristics of childhood trauma in our samples of pregnant women and expecting men from the community.

## Methods

### Study design and sample

A sample of 2853 pregnant women (*M*_*age*_ = 29.36, SD = 4.31, range = 18-46) and a sample of 561 expecting men (*M*_*age*_ = 30.84, SD = 5.41, range = 20-55) were recruited in the province of Quebec, Canada using three complementary strategies. The first strategy permitted us to recruit 886 women and 425 men. These participants were invited to participate in the study during prenatal classes between July 2015 and September 2018. They reported on their history of trauma but did not complete measures of psychiatric symptoms. The second strategy had us recruit 630 women and 117 men. These participants were informed of the study at their first pregnancy monitoring appointment between April 2018 and March 2021. Those who agreed to participate were subsequently contacted during the second trimester of pregnancy and invited to complete the full set of measures. The third strategy permitted us to recruit 1337 women and 19 men. These participants were recruited online, through social media, between April 2nd and April 13th, 2020. Sociodemographic characteristics for the three sets of participants are reported in Tables S1 and S2. Inclusion criteria were being 18 years old or older, having sufficient reading skills to complete self-reported instruments and being pregnant/awaiting a child. There were no exclusion criteria based on psychiatric diagnoses. Studies received ethical approval from the Comité d’éthique de la recherche avec des êtres humains de l’Université du Québec à Trois-Rivières and the Comité d’éthique de la recherche du Centre intégré universitaire de santé et de services sociaux de la Mauricie-et-du-Centre-du-Québec.

### Measures

#### Childhood Trauma Questionnaire

Childhood trauma was assessed using the French version [24] of the Childhood Trauma Questionnaire (CTQ-28) [25]. The 28-item self-reported measure examines five types of childhood trauma: physical, emotional, and sexual abuse as well as physical and emotional neglect. Responses to each item are rated on a 5-point Likert scale, ranging from 1 (*never true*) to 5 (*very often true*). Cut-offs are validated for each subscale (physical abuse ≥8, emotional abuse ≥ 10, sexual abuse ≥ 8, physical neglect ≥ 8 and emotional neglect ≥ 15) [26]. In the current study, participants with at least one subscale with a score above the cut-off were classified as having been exposed to childhood trauma, whereas cumulative childhood trauma was defined as having been exposed to at least two different types of trauma. The CTQ-28 shows a good validity across diverse clinical and general populations [25]. The Cronbach’s alpha for the CTQ in this study was α = .82.

#### PTSD Checklist for DSM-5

Post-traumatic stress symptoms were assessed using the validated French version [27] of the *PTSD Checklist for DSM-5* (PCL-5) [28]. This 20-item self-reported questionnaire is based on the PTSD diagnostic criteria of the DSM-5. Responses are rated on a 5-point Likert scale ranging from 0 (*not at all*) to 4 (*always*). Higher scores reflect greater severity of symptoms and a score at or higher than 33 would be highly predictive of a DSM-V diagnosis of PTSD [29]. Both the French and the English versions have good reliability (internal consistency, temporal stability, test-retest) and convergent validity [27–29]. The Cronbach’s alpha for the PCL-5 in this study was α = .92.

#### Kessler psychological distress scale

Anxiety and depression were measured using the French version [30] of the 10-item *Kessler Psychological Distress Scale* (K10) [31]. Responses are rated on a 5-point Likert scale ranging from 1 (*none of the time*) to 5 (*all of the time*). We used a clinical cut-off of 30 as 76.3% of adults with scores ≥ 30 would meet the diagnostic criteria for an anxiety, affective or substance use disorder [32]. The instrument is adequate for screening mood and anxiety disorders in pregnant women [33]. The Cronbach’s alpha for the K-10 in this study was α = .88.

#### State-trait anger expression inventory-2

Current intensity of angry feelings and expression of anger was assessed using the State Anger scale of the *State-Trait Anger Expression Inventory-2* (STAXI-2) [34]. This scale contains 15 items, and responses are rated on a 4-point Likert scale ranging from 1 (*almost never*) to 4 (*almost always*). Scores higher than two standard deviations (*T* scores of 70) in Spielberger’s normative sample of women (> 28) and men (> 32) aged between 20 and 29 years were here considered as a level of anger interfering with functioning [34]. The Cronbach’s alpha for the State Anger scale in this study was α = .93.

#### Self and interpersonal functioning scale

Global personality impairment was assessed using the 24-item *Self and Interpersonal Functioning Scale* (SIFS) [35]. Its items are rated on a 5-point Likert scale ranging from 0 (*this does not describe me at all*) to 4 (*this totally describes me*), higher scores indicating higher dysfunction. We used a clinical cut-off of ≥ 1.90 which reflects moderate severity personality disorders [36]. The SIFS shows good validity indices across samples [35, 37, 38]. The Cronbach’s alpha for the SIFS in this study was α = .86.

### Data analyses

Data analyses were performed using the Statistical Package for Social Sciences, version 27.0. Data screening for normality detected univariate outliers (standardized scores over 3.29). To reduce their impact on the distribution, we replaced outliers by a raw score of one unit larger than the next highest score in the distribution [39]. Mahalanobis distances showed no further multivariate outliers, and variables were normally distributed after this transformation.

Descriptive analyses were first performed to determine the proportion of adults who experienced childhood trauma. All analyses were performed separately for men and women. Chi-squares were next used to evaluate whether childhood trauma varied as a function of individual or sociodemographic variables. Complementary continuous and categorical approaches were used to evaluate whether childhood trauma was associated with complex psychological symptoms. For the continuous approach, we computed a *symptom complexity score* by adding the standardized scores of the four measures of psychiatric symptoms. A Pearson correlation was first performed to evaluate the association between the number of childhood trauma and the complexity of psychiatric problems. We next evaluated whether childhood trauma and complexity of symptoms shared variance that could not be accounted for by other risk factors by performing a hierarchical regression analysis including four sociodemographic variables (age, education, familial income, and marital status) in a first set of predictors, and childhood trauma in a second step. For the categorical approach, we computed a score of *comorbid psychiatric problems* by adding the number of psychiatric symptoms reaching the clinical cut-offs of the instruments. It is noteworthy that we used severe cut-offs for each instrument to ensure that participants were highly likely to have a psychiatric diagnosis. We used chi-square analyses to evaluate the distribution of comorbid psychiatric problems in participants without trauma, single trauma, and cumulative trauma. We also calculated the relative risk (RR) of presenting comorbid problems in participants exposed to a single type of trauma and to cumulative trauma while using participants without trauma as the reference group.

## Results

Sociodemographic data for women and men are presented in Table 1. Overall, participants were mainly married or in common-law relationships (96.8%), Caucasian (95.9%) and had some post-secondary education (90.7%). Median household income was between C$85 000 and C$95 000. Our sample of expecting adults (mainly women) is thus representative of mothers’ from the Province of Quebec where 91.5% of women between 25 and 34 years have post-secondary education [40] and the median income for households of two adults and one child is C$93 400 [41]. Analyses on symptom complexity were performed on a subsample of 1779 women and 118 men with complete data. Participants who did not complete the full data set (data on childhood trauma only) were not different from participants who completed all measures in terms of exposure to trauma (respectively 32.4% and 34.8%), *χ*^2^(1) = 2.22, *p* = .14.

**Table 1:** Demographic characteristics of participants

	Participants included in the analyses on prevalence only		Participants included in the analyses on symptom complexity	
Demographics	Women (n = 2224)	Men (n = 212)	Women (n = 1779)	Men (n = 118)
**Age**, mean (SD)	29.4 (4.3)	30.8 (5.4)	29.52 (4.20)	31.55 (6.04)
**Primiparous**, n (%)	1425 (65.7%)	127 (68.3%)	1131 (63.7%)	65 (55.1%)
**Marital status**, n (%)				
In relationship	2145 (96.8%)	212 (100%)	1717 (96.8%)	118 (100%
Single	72 (3.25%)	0	56 (3.2%)	-
**Education level**, n (%)				
No high school diploma	58 (2.8%)	17 (8.0%)	31 (1.7%)	8 (6.8%)
High school diploma	125 (5.6%)	28 (13.2%)	97 (5.5%)	14 (11.9%)
Collegial or professional training	930 (41.8%)	106 (50.0%)	726 (40.9%)	118 (49.2%)
University degree	1107 (49.8%)	61 (28.8%)	921 (51.9%)	38 (32.2%)
**Ethnicity**, n (%)				
White	2109 (95.9%)	199 (95.7%)	1698 (96.4%)	113 (96.6%)
First Nations	19 (0.9%)	3 (1.4%)	10 (0.6%)	1 (0.9%)
Black	19 (0.9%)	1 (0.5%)	13 (0.7%)	1 (0.9%)
Hispanic	25 (1.1%)	2 (1.0%)	22 (1.2%)	2 (1.7%)
Asian	8 (0.4%)	3 (1.4%)	5 (0.3%)	0
Other	19 (0.9%)	0	14 (0.8%)	0
**Annual household income**, n (%)				
Can $34 999 or less	182 (8.7%)	20 (14.0%)	147 (8.3%)	14 (11.9%)
Can $35 000 – 64 999$	375 (18.0%)	31 (21.7%)	304 (17.2%)	26 (22.0%)
Can $65 000$ - 85 999$	656 (31.5%)	49 (34.3%)	547 (30.9%)	42 (35.6%)
Can $95 000 or more	869 (41.7%)	34 (30.1%)	751 (42.5%)	36 (30.5%)

*Note.* Sociodemographic data were available for 2436 participants (2224 women).

### Trauma characteristics and prevalence

Thirty-five percent of pregnant women (n = 999) and 28% of expecting men (n = 156) reported having been exposed to at least one type of trauma during childhood. As shown in Table 2, the most prevalent types of trauma were physical neglect and emotional abuse. Half of the women (49.2%) and 44% of the men with a history of trauma experienced multiple types of trauma. Women were significantly more likely to report childhood trauma (35.0%) than men (27.8%), particularly under the forms of sexual and emotional abuse.

**Table 2:** Prevalence of childhood trauma in pregnant women and expecting men

Variables	Women (n = 2853) n (%)	Men (n = 561) n (%)	Group differences (women vs men) *p*-value^1^
Any type of trauma	999 (35.0)	156 (27.8)	.001
Single trauma	508 (17.8)	88 (14.8)	.002
Cumulative trauma	491 (17.2)	68 (12.1)	
Physical abuse	224 (7.9)	50 (8.9)	.40
Sexual abuse	330 (11.6)	18 (3.2)	< .001
Emotional abuse	543 (19.0)	75 (13.4)	.001
Emotional neglect	305 (10.7)	44 (7.8)	.04
Physical neglect	552 (19.3)	97 (17.3)	.26

^1^Given the 5 types of trauma, we corrected for multiple analyses using Bonferroni correction by dividing the significance level of 0.05 by 5. The significance level was then fixed at *p* = 0.01.

Reports of trauma were more frequent in multiparous (38.6%) than in primiparous (31.7%) parents, *χ*^2^(1) = 14.25, *p* < .001, but did not change according to the trimester of pregnancy during which it had been assessed, *χ*^2^(2) = 0.84, *p* = .66. Prevalence of trauma was higher among participants reporting current sociodemographic risk factors, including being younger than twenty years old at the beginning of pregnancy [73.7% vs 33.8%; *χ*^2^(1) = 13.30, *p* < .001], having an income inferior to the low-income cut-off [55.0% vs 32.7%; *χ*^2^(1) = 40.22, *p* < .001], not having completed high school [53.3% vs 33.6%, *χ*^2^(1) = 12.64, *p* < .001] and being a single parent [62.0% vs 33.2%, *χ*^2^(1) = 28.17, *p* < .001]. Thirty-four percent (34.1%, n = 77) of women and 28.6% of men (n = 8) reporting at least one sociodemographic risk factor had experienced cumulative childhood trauma, significantly higher rates than women [15.0%, n = 277; *χ*^2^(2) = 63.75, *p* < .001] and men [10.4%, n = 12; *V*(2) = 0.21, *p* = .04] not reporting any risk factor.

### Cumulative childhood trauma, symptom complexity and comorbid psychiatric problems

**Fig. 1:**
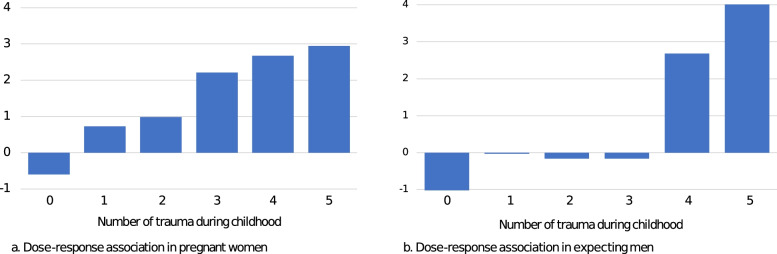
*Dose-response association between number of traumas in childhood and complexity of psychiatric symptoms in pregnant women and expecting men.* The score of symptom complexity was computed by adding the standardized scores of the four measures of psychiatric symptoms (PTSD, psychological distress, state anger and personality impairment). Number of traumas during childhood was assessed by adding scales reaching the cut-offs of the Childhood Trauma Questionnaire (physical, emotional, and sexual abuse, physical and emotional neglect)

A dose-response relationship between the number of traumas experienced during childhood and the complexity of psychiatric symptoms during pregnancy was observed in women (r = 0.31, *p* < .001) and men (r = 0.44, *p* < .001) (Figure 1). Hierarchical regression analyses showed that childhood trauma significantly improved the prediction of symptom complexity over the effect of sociodemographic risk factors (Table 3) in both women (Δ*R*^*2*^ = 8.3%) and men (Δ*R*^*2*^ = 22.2%).

**Table 3:** Hierachical multiple regressions on the predictive role of childhood trauma on symptom complexity over the effect of sociodemographic risk factors for women and men

Criteria and predictors	Model 1			Model 2		
	*B*	*SE*	*β*	*B*	*SE*	*β*
**Women**						
(Constant)	2.52	0.56		-0.51	0.58	
Annual income	-0.21***	0.03	-0.16	-0.15***	0.03	-0.12
Marital status	-0.003	0.05	-0.001	-0.02	0.05	-0.01
Age	0.03	0.02	0.04	-0.01	0.02	-0.01
Education level	-0.36***	0.07	-0.15	-0.21**	0.07	-0.08
Severity of trauma				0.09***	0.01	0.30
*R*^*2*^	0.07			0.15		
*F*	30.34***			60.55***		
∆*R*^*2*^				0.08		
**Men**						
(Constant)	0.56	1.62		-3.59	1.59	
Annual income	-0.25*	0.12	-0.24	-0.18	0.10	-0.16
Marital status	0.06	0.22	0.03	-0.06	0.19	-0.03
Age	0.01	0.04	0.01	-0.03	0.04	-0.06
Education level	0.03	0.21	0.02	0.24	0.19	0.12
Severity of trauma				0.12***	0.02	0.49
*R*^*2*^	0.05			0.27		
*F*	1.41			8.11***		
∆*R*^*2*^				0.22		

Note. * p < .05. ** *p* < .01. *** *p* < .001. Model 1 included all sociodemographic indices (annual income, marital status, age, education level) as predictors. Severity of trauma during childhood was added as a predictor in Model 2.

Analyses using categorical scores yielded similar results. Women and men exposed to cumulative childhood trauma had a greater probability of experiencing comorbid problems during pregnancy (respectively 15.2% and 18.8%) than adults who experienced a single type of trauma (9.8% of women and no men) or no trauma (3.4% of women and no men) (Table 4). The relative risk of presenting comorbid problems during pregnancy was of 2.78 in women who experienced one type of trauma and of 4.95 in women with cumulative trauma, in comparison to women without trauma.Relative risks for men could not be estimated given the small sample size and the absence of men presenting comorbid psychiatric problems without a history of trauma.

**Table 4:** Distributions of psychiatric comorbidities during pregnancy according to the level of exposure to childhood trauma

	No trauma		One trauma		Cumulative trauma^**2**^	
Psychiatric problems^**1**^	Freq	%	Freq	%	Freq	%
**Women**^**3**^						
No psychiatric problem	1010	69.2%	244	16.7%	205	14.1%
One psychiatric problem	97	50.3%	45	23.3%	51	26.4%
Comorbid psychiatric problems	39	34.5%	28	24.8%	46	40.7%
**Men**^**4**^						
No psychiatric problem	73	71.6%	17	16.7%	12	11.8%
One psychiatric problem	7	63.6%	3	27.3%	1	9.1%
Comorbid psychiatric problems	0	0%	0	0%	3	100%

^1^Groups of psychiatric problems were computed according to the clinical cut-offs of the PCL-5 (≥ 33), K10 (≥ 30), STAXI-2 (*T* scores > 70), and SIFS (≥1.90).

^2^Groups of trauma were computed using the cut-offs of the Childhood Trauma Questionnaire. Participants were considered as having experienced multiple trauma when the scored higher than the cut-off on at least two scales among physical abuse (≥ 8), emotional abuse (≥ 10), sexual abuse (≥ 8), physical neglect (≥ 8), and emotional neglect (≥ 15).

^3^Women having been exposed to cumulative childhood trauma were significantly more likely to report comorbid problems during pregnancy than women who experienced a single type or no trauma, *χ*^2^(4) = 88.44, *p* < .001.

^4^Men having been exposed to cumulative childhood trauma were significantly more likely to report comorbid problems during pregnancy than men who experienced a single type or no trauma, *V*(4) = 0.29, *p* < .001.

## Discussion

Our study examined the association between cumulative childhood trauma and symptom complexity in pregnant women and expecting men. Our findings showed a dose-response relationship between the number of different traumas pregnant women and expecting men had been exposed to during their childhood and the accumulation of symptoms belonging to different psychiatric problems, including symptoms of PTSD, anxiety, depression, anger and personality disorders. The variance in psychiatric symptomatology explained by childhood trauma was more important than the variance explained by other important sociodemographic risk factors, namely teen pregnancy, having a low income, not having completed high school and being a single parent. Our results using thresholds previously validated against psychiatric diagnoses additionally revealed that pregnant women having been exposed to single and cumulative trauma during childhood were respectively at a 2.78- and 4.95-times greater risk of presenting comorbid psychiatric problems during pregnancy, in comparison to women without trauma. However, a small proportion of women without childhood trauma also reported comorbidities, suggesting that trauma is a powerful enhancer of the aggregation of psychiatric problems, but not a necessary condition.

These results are in line with various studies showing that trauma affects multiple spheres of functioning over the sole presence of PTSD symptoms [13–15, 17, 18]. For instance, Briere and colleagues first observed, in 2453 female university students, a linear relationship between cumulative childhood trauma and symptom complexity, operationalized by the number of clinical scales exceeding the threshold [13]. Cloitre and colleagues also reported that each additional type of trauma experienced during childhood increased symptom complexity in adulthood by 17% [14]. Finally, Steine and colleagues similarly revealed a significant dose-response relation between cumulative childhood trauma and a measure of symptom complexity based on self-reported symptoms of PTSD, anxiety, depression, eating disorders, insomnia, and physical and emotional pain amongst adult survivors of sexual abuse [15]. However, none of these studies had investigated the association between cumulative childhood trauma and symptom complexity during the antenatal period.

We also took the opportunity provided by our study to estimate the rate of self-reported childhood trauma in a large sample of pregnant women and expecting men from the community. Our findings showed that childhood trauma is highly prevalent, with 35% of pregnant women and 28% of expecting men in our sample reporting at least one type of abuse or neglect according to the validated cut-offs of the Childhood Trauma Questionnaire. This rate is similar to what has been reported in non-pregnant samples of adults using the same instrument [2]. Furthermore, our prevalence rates are representative of those obtained from large population-based studies which generally reported that around a third of adults from the general population experienced childhood trauma [1, 2]. Moreover, our study adds to the small body of knowledge about trauma in expecting fathers. We observed that expecting fathers were less likely to report having experienced sexual and emotional abuse than pregnant women, resulting in lower rates of exposure to trauma and cumulative trauma. Despite these lower reports, the size of the association between cumulative trauma and the complexity of psychiatric symptoms during the antenatal period was slightly larger in men than in women. The higher prevalence of sexual abuse [42] and cumulative trauma [1] in women as well as the association between trauma and complex psychopathology in expecting men [43] is consistent with previous studies.

Our findings that trauma is frequent in expecting parents from the community and that cumulative trauma drastically increases the risk of presenting complex psychiatric symptoms during pregnancy have implications for scientific research and perinatal practices. Indeed, a history of trauma would negatively impact the experience of parenthood [6, 44] and the quality of parenting [45]. A recent synthesis of meta-analyses also revealed that parental experiences of childhood trauma would figure amongst the most important risk factors for childhood maltreatment [46]. Cumulative childhood trauma would further increase the risk of continuity of maltreatment through the next generation [47]. Maternal childhood trauma has also been associated with a variety of adverse child outcomes such as preterm birth and low birth weight [48], difficult temperament [49], developmental delays [50], emotional and behavioral difficulties [51], poor overall health [52] and insecure or disorganized attachment [53]. Importantly, maternal mental health would be a crucial mechanism linking exposure to trauma in mothers to poor maternal confidence and antenatal attachment [6], pregnancy complications [54] as well as to poor child outcomes [55]. Our findings suggest that pregnant women and expecting men who experienced significant adversity during their childhood should be considered as particularly vulnerable and should be closely monitored. Innovative interventions with this population may be necessary [56] considering that comorbid disorders, as well as disorders that arise from a history of childhood trauma, would be more resistant to treatment [21]. To date, only a few evidence-based antenatal interventions have been designed to address the specific needs and challenges of pregnant women with histories of childhood trauma [57–59] but it remains to be determined whether or not such psychological interventions contribute to lowering the complex psychiatric symptomatology frequently encountered in this population.

The results of our study should be interpreted in light of some limitations. First, due to the correlational nature of our study, no causal association between our variables can be inferred. Second, the evaluation of childhood trauma was retrospective, which is prone to recall bias. In this regard, a recent study reported inconsistencies in retrospective reports of childhood abuse across the perinatal period, which could be partly explained by psychosocial risk factors [3]. Third, self-reported instruments were used to assess psychological symptoms. Future studies should consider using standardized clinical interviews to increase the validity of the findings. Fourth, we found no consensus on how to measure the concept of symptom complexity. Our approach has the advantage of capturing a large spectrum of symptoms, including externalized problems, internalized problems, trauma-related symptoms, and personality dysfunctions, as recommended in a recent research on symptom complexity [19]. In the absence of a common definition of symptom complexity, comparison of findings across studies will remain limited. Fifth, lifetime traumas were not taken into account, whereas some studies have found that traumatic experiences in childhood and adulthood can have cumulative effects on mental health [60]. Finally, our study included a minority of men, but given the paucity of data on expecting men, it seemed appropriate to include them. However, the comparisons between men and women should be interpreted with caution.

## Conclusions

Overall, this study showed that the accumulation of traumatic experiences during childhood and adolescence is associated with complex psychiatric symptoms during pregnancy in men and women, over and beyond the effect of sociodemographic risk factors. These symptoms may in turn compromise the experience of parenting and the development of the child. Since our results highlighted that such trauma is highly frequent in the general population of pregnant women and expecting men, the inclusion of the paradigm of trauma-informed care in obstetric and gynecologic practices appears well justified. Such practices should recognize the impact of trauma and understand paths for recovery, recognize the signs and symptoms of trauma, respond by incorporating trauma knowledge into policies and practices, and resist actively to re-traumatization [61].

## Supplementary Information

**Additional file 1.**

## Data Availability

The datasets used and/or analyzed during the current study are available from the corresponding author on reasonable request.

## List of abbreviations

PTSD: post-traumatic stress disorder

## References

[1] Afifi TO, MacMillan HL, Boyle M, Taillieu T, Cheung K, Sareen J (2014). Child abuse and mental disorders in Canada. CMAJ..

[2] Witt A, Brown RC, Plener PL, Brähler E, Fegert JM. Child maltreatment in Germany: prevalence rates in the general population. Child Adolesc Psychiatry Ment Health. 2017;11(1):1-9. 10.1186/s13034-017-0185-0 PMC5621113 28974983

[3] Racine N, Plamondon A, McDonald S, Tough S, Madigan S (2020). The consistency of maternal childhood abuse reporting in pregnancy and the postpartum period. J Womens Health..

[4] Seng JS, D'Andrea W, Ford JD (2014). Complex mental health sequelae of psychological trauma among women in prenatal care. Psychol Trauma..

[5] Sørbø MF, Grimstad H, Bjørngaard JH, Schei B, Lukasse M (2013). Prevalence of sexual, physical and emotional abuse in the Norwegian mother and child cohort study. BMC Public Health..

[6] Berthelot N, Lemieux R, Garon-Bissonnette J, Muzik M (2020). Prenatal Attachment, Parental Confidence, and Mental Health in Expecting Parents: The Role of Childhood Trauma. J Midwifery Womens Health..

[7] Li Y, Long Z, Cao D, Cao F (2017). Maternal history of child maltreatment and maternal depression risk in the perinatal period: A longitudinal study. Child Abuse Negl..

[8] McDonnell CG, Valentino K (2016). Intergenerational effects of childhood trauma: Evaluating pathways among maternal ACEs, perinatal depressive symptoms, and infant outcomes. Child Maltreat..

[9] Muzik M, McGinnis EW, Bocknek E, Morelen D, Rosenblum KL, Liberzon I (2016). PTSD symptoms across pregnancy and early postpartum among women with lifetime PTSD diagnosis. Depress Anxiety..

[10] Racine N, Devereaux C, Cooke JE, Eirich R, Zhu J, Madigan S (2021). Adverse childhood experiences and maternal anxiety and depression: a meta-analysis. BMC Psychiatry..

[11] Choi KW, Sikkema KJ (2016). Childhood maltreatment and perinatal mood and anxiety disorders: A systematic review. Trauma Violence Abuse..

[12] Meltzer-Brody S, Larsen J, Petersen L, Guintivano J, Florio AD, Miller W (2018). Adverse life events increase risk for postpartum psychiatric episodes: A population-based epidemiologic study. Depress Anxiety..

[13] Briere J, Kaltman S, Green BL (2008). Accumulated childhood trauma and symptom complexity. J Trauma Stress..

[14] Cloitre M, Stolbach BC, Herman JL, van der Kolk B, Pynoos R, Wang J (2009). A developmental approach to complex PTSD: childhood and adult cumulative trauma as predictors of symptom complexity. J Trauma Stress..

[15] Steine IM, Winje D, Krystal JH, Bjorvatn B, Milde AM, Grønli J (2017). Cumulative childhood maltreatment and its dose-response relation with adult symptomatology: Findings in a sample of adult survivors of sexual abuse. Child Abuse Negl..

[16] Berthelot N, Garon-Bissonnette J, Jomphe V, Doucet-Beaupré H, Bureau A, Maziade M. Childhood trauma may increase the risk of psychosis and mood disorder in genetically high-risk children and adolescents by enhancing the accumulation of risk indicators. Schizophr Bull Open. Submitted. . 10.1093/schizbullopen/sgac017 PMC11206050 39144791

[17] Hodges M, Godbout N, Briere J, Lanktree C, Gilbert A, Kletzka NT (2013). Cumulative trauma and symptom complexity in children: A path analysis. Child Abuse Negl..

[18] Putnam KT, Harris WW, Putnam FW (2013). Synergistic childhood adversities and complex adult psychopathology. J Trauma Stress..

[19] Bigras N, Godbout N, Hébert M, Sabourin S (2017). Cumulative adverse childhood experiences and sexual satisfaction in sex therapy patients: What role for symptom complexity?. J Sex Med..

[20] Anastas JW, Payne NA, Ghuman SJ (2021). Adverse Childhood Experiences and Complex Post-traumatic Stress in Pregnant Teens: A Pilot Study. Matern Child Health J..

[21] Coventry P, Meader N, Melton HA, Temple M, Dale H, Wright K, et al. Psychological and pharmacological interventions for PTSD and comorbid mental health problems following complex traumatic events: systematic review and component network meta-analysis. PLoS Med. 2020. 10.1371/journal.pmed.1003262 PMC7446790 32813696

[22] Karatzias T, Murphy P, Cloitre M, Bisson J, Roberts N, Shevlin M (2019). Psychological interventions for ICD-11 complex PTSD symptoms: systematic review and meta-analysis. Psychol Med..

[23] Saris I, Aghajani M, van der Werff S, Van der Wee N, Penninx B (2017). Social functioning in patients with depressive and anxiety disorders. Acta Psychiatr Scand..

[24] Lacharité C, Deshaulniers R, St-Laurent D. Le questionnaire des traumatismes vécus en enfance. Traduction française du Childhood Trauma Questionnaire. 2002.

[25] Bernstein DP, Stein JA, Newcomb MD, Walker E, Pogge D, Ahluvalia T (2003). Development and validation of a brief screening version of the Childhood Trauma Questionnaire. Child Abuse Negl..

[26] Walker EA, Unutzer J, Rutter C, Gelfand A, Saunders K, VonKorff M (1999). Costs of health care use by women HMO members with a history of childhood abuse and neglect. Arch Gen Psychiatry..

[27] Ashbaugh AR, Houle-Johnson S, Herbert C, El-Hage W, Brunet A (2016). Psychometric Validation of the English and French Versions of the Posttraumatic Stress Disorder Checklist for DSM-5 (PCL-5). PLoS One..

[28] Wilkins KC, Lang AJ, Norman SB (2011). Synthesis of the psychometric properties of the PTSD checklist (PCL) military, civilian, and specific versions. Depress Anxiety..

[29] Bovin MJ, Marx BP, Weathers FW, Gallagher MW, Rodriguez P, Schnurr PP (2016). Psychometric properties of the PTSD Checklist for Diagnostic and Statistical Manual of Mental Disorders–Fifth Edition (PCL-5) in veterans. Psychol Assess..

[30] Gravel R, Connolly D, Bédard M. Enquête sur la santé dans les collectivités canadiennes : santé mentale et bien-être. Statistics Canada; 2002.

[31] Kessler RC, Andrews G, Colpe LJ, Hiripi E, Mroczek DK, Normand SLT (2002). Short screening scales to monitor population prevalences and trends in non-specific psychological distress. Psychol Med..

[32] Andrews G, Slade T (2001). Interpreting scores on the Kessler Psychological Distress Scale (K10). Australian and New Zealand Journal of Public Health..

[33] Spies G, Stein DJ, Roos A, Faure SC, Mostert J, Seedat S (2009). Validity of the Kessler 10 (K-10) in detecting DSM-IV defined mood and anxiety disorders among pregnant women. Arch Womens Ment Health..

[34] Spielberger CD (1999). STAXI-2: State-Trait Anger Expression Inventory-2. Professional Manual.

[35] Gamache D, Savard C, Leclerc P, Côté A (2019). Introducing a short self-report for the assessment of DSM–5 level of personality functioning for personality disorders: The Self and Interpersonal Functioning Scale. Personal Disord..

[36] Gamache D, Savard C, Leclerc P, Payant M, Berthelot N, Côté A, et al. A Proposed Classification of ICD-11 Severity Degrees of Personality Pathology Using the Self and Interpersonal Functioning Scale. Front. Psychiatry. 2021;12(292). 10.3389/fpsyt.2021.628057 PMC8012561 33815167

[37] Waugh MH, McClain CM, Mariotti EC, Mulay AL, DeVore EN, Lenger KA (2021). Comparative content analysis of self-report scales for level of personality functioning. J Pers Assess..

[38] Gamache D, Savard C, Lemieux R, Berthelot N. Impact of level of personality pathology on affective, behavioral, and thought problems in pregnant women during the coronavirus disease 2019 pandemic. Personal Disord. 2021. 10.1037/per0000479 33411559

[39] Tabachnick BG, Fidell LS (2013). Using Multivariate Statistics.

[40] Canada S. Education Highlight Tables 2016 Census. 2016.

[41] Québec Idlsd. Revenu médian à l’échelle du Québec : Revenu médian, revenu total, ménages, Québec, 1996-2018. In: Québec Gd, editor. 2018.

[42] Stoltenborgh M, Bakermans-Kranenburg MJ, Alink LR, van IJzendoorn MH. (2015). The prevalence of child maltreatment across the globe: Review of a series of meta-analyses. Child Abuse Rev..

[43] Skjothaug T, Smith L, Wentzel-Larsen T, Moe V (2015). Prospective fathers’ adverse childhood experiences, pregnancy-related anxiety, and depression during pregnancy. Infant Ment Health J..

[44] Chamberlain C, Gee G, Harfield S, Campbell S, Brennan S, Clark Y, et al. Parenting after a history of childhood maltreatment: A scoping review and map of evidence in the perinatal period. PLoS One. 2019;14(3):e0213460-e. 10.1371/journal.pone.0213460 PMC6415835 30865679

[45] Savage L-É, Tarabulsy GM, Pearson J, Collin-Vézina D, Gagné L-M (2019). Maternal history of childhood maltreatment and later parenting behavior: A meta-analysis. Dev Psychopathol..

[46] Ijzendoorn MH, Bakermans-Kranenburg MJ, Coughlan B, Reijman S (2020). Annual research review: Umbrella synthesis of meta-analyses on child maltreatment antecedents and interventions: Differential susceptibility perspective on risk and resilience. J Child Psychol Psychiatry..

[47] St-Laurent D, Dubois-Comtois K, Milot T, Cantinotti M (2019). Intergenerational continuity/discontinuity of child maltreatment among low-income mother–child dyads: The roles of childhood maltreatment characteristics, maternal psychological functioning, and family ecology. Dev Psychopathol..

[48] Nesari M, Olson JK, Vandermeer B, Slater L, Olson DM (2018). Does a maternal history of abuse before pregnancy affect pregnancy outcomes? A systematic review with meta-analysis. BMC Pregnancy Childbirth..

[49] Bouvette-Turcot A-A, Fleming AS, Unternaehrer E, Gonzalez A, Atkinson L, Gaudreau H, et al. Maternal symptoms of depression and sensitivity mediate the relation between maternal history of early adversity and her child temperament: The inheritance of circumstance. Dev Psychopathol. 2019. 10.1017/S0954579419000488 31156070

[50] Garon-Bissonnette J, Duguay G, Lemieux R, Dubois-Comtois K, Berthelot N. Maternal childhood abuse and neglect predicts offspring development in early childhood: The roles of reflective functioning and child sex. Child Abuse Negl. 2021. 10.1016/j.chiabu.2021.105030 33752901

[51] Plant DT, Jones FW, Pariante CM, Pawlby S (2017). Association between maternal childhood trauma and offspring childhood psychopathology: mediation analysis from the ALSPAC cohort. The British journal of psychiatry : the journal of mental science..

[52] Lê-Scherban F, Wang X, Boyle-Steed KH, Pachter LM. Intergenerational Associations of Parent Adverse Childhood Experiences and Child Health Outcomes. Pediatrics. 2018;141(6). 10.1542/peds.2017-4274 29784755

[53] Berthelot N, Ensink K, Bernazzani O, Normandin L, Luyten P, Fonagy P (2015). Intergenerational transmission of attachment in abused and neglected mothers: the role of trauma-specific reflective functioning. Infant Ment Health J..

[54] Yampolsky L, Lev-Wiesel R, Ben-Zion IZ (2010). Child sexual abuse: is it a risk factor for pregnancy?. J Adv Nurs..

[55] Alto ME, Warmingham JM, Handley ED, Rogosch F, Cicchetti D, Toth SL (2021). Developmental pathways from maternal history of childhood maltreatment and maternal depression to toddler attachment and early childhood behavioral outcomes. Attach Hum Dev..

[56] Berthelot N, Garon-Bissonnette J, Lemieux R, Drouin-Maziade C, Maziade M (2020). Paucity of intervention research in childhood maltreatment contrasts with the long known relation with mental health disorders: Is trauma research translational enough?. Ment Health Prev..

[57] Berthelot N, Lemieux R, Lacharité C (2018). Development of a prenatal program for adults with personal histories of childhood abuse or neglect: a Delphi consensus consultation study. Health Promot Chronic Dis Prev Can..

[58] Lieberman AF, Diaz MA, Castro G, Oliver BG (2020). Make Room for Baby: Perinatal Child-Parent Psychotherapy to Repair Rrauma and Promote Attachment.

[59] Seng J, Sperlich M, Rowe H, Cameron H, Harris A, Rauch SA (2011). The Survivor Moms’ Companion: Open pilot of a posttraumatic stress specific psychoeducation program for pregnant survivors of childhood maltreatment and sexual trauma. IJCB..

[60] Seng JS, Sperlich M, Low LK (2008). Mental health, demographic, and risk behavior profiles of pregnant survivors of childhood and adult abuse. J Midwifery Womens Health..

[61] SAMHSA. SAMHSA’s Concept of Trauma and Guidance for a Trauma-Informed Approach. In: Initiative TaJS, editor.: Department of Health & Human Services USA; 2014.

