# Supplementary material for: Cumulative childhood trauma and complex psychiatric symptoms in pregnant women and expecting men
Source: BMC Pregnancy Childbirth. 2022 Jan 4;22:10. doi: 10.1186/s12884-021-04327-x (PMC8725451; doi:10.1186/s12884-021-04327-x)
Supplement: Supplementary file 1 — Additional file 1. [file 12884_2021_4327_MOESM1_ESM.docx]

**Supplemental information to the manuscript “Cumulative Childhood Trauma and Complex Psychiatric Symptoms in Pregnant Women and Expecting Men”**

*BMC Pregnancy & Childbirth*

Table of contents

[**Table S1.** Characteristics of participants included in the analyses on the prevalence of trauma according to recruitment strategy 2](#_Toc54092368)

**Table S2.** Characteristics of the subsample of participants included in the analyses on symptom complexity according to recruitment strategy 3

**Table S1.** Characteristics of participants included in the analyses on the prevalence of trauma according to recruitment strategy

| Demographics | First strategy - Prenatal classes  (n = 336) | Second strategy – Medical appointment  (n = 747) | Third strategy – Online recruitment  (n = 1353) |
| --- | --- | --- | --- |
| **Age**, mean (SD) | 28.6 (4.5) | 29.6 (5.1) | 29.7 (4.0) |
| **Sex**, n (%) |  |  |  |
| Women | 260 (77.4%) | 630 (84.3%) | 1334 (98.6%) |
| Men | 76 (22.6%) | 117 (15.7%) | 19 (1.4%) |
| **Primiparous**, n (%) | 244 (90.4%) | 351 (47.8%) | 244 (70.8%) |
| **Marital status**, n (%) |  |  |  |
| In relationship | 230 (95.5%) | 708 (94.9%) | 1322 (98.1%) |
| Single | 15 (4.5%) | 38 (5.1%) | 26 (1.9%) |
| **Education level**, n (%) |  |  |  |
| No high school diploma | 22 (6.5%) | 42 (5.6%) | 11 (0.8%) |
| High school diploma | 25 (7.4%) | 62 (8.3%) | 66 (4.9%) |
| Collegial or professional training | 157 (46.7%) | 371 (49.8%) | 508 (37.6%) |
| University degree | 132 (39.3%) | 270 (36.2%) | 766 (56.7%) |
| **Ethnicity**, n (%) |  |  |  |
| White | 308 (95.9%) | 696 (94.7%) | 1304 (96.9%) |
| First Nations | 4 (1.2%) | 9 (1.2%) | 9 (0.7%) |
| Black | 3 (0.9%) | 12 (1.6%) | 5 (0.4%) |
| Hispanic | 2 (0.6%) | 12 (1.6%) | 5 (0.4%) |
| Asian | 4 (1.2%) | 1 (0.1%) | 6 (0.4%) |
| Other | 5 (1.5%) | 5 (0.7%) | 9 (0.7%) |
| **Annual household income**, n (%) |  |  |  |
| Can $34 999 or less | 16 (10.1%) | 110 (14.0%) | 76 (5.7%) |
| Can $35 000 – 64 999$ | 45 (28.5%) | 164 (22.3%) | 197 (14.8%) |
| Can $65 000$ - 85 999$ | 49 (31.0%) | 240 (32.6%) | 416 (31.3%) |
| Can $95 000 or more | 48 (30.4%) | 222 (30.2%) | 642 (48.2%) |
| **Prevalence of trauma**, n (%) |  |  |  |
| No trauma | 236 (70.2%) | 474 (63.5%) | 892 (65.9%) |
| One trauma | 50 (14.9%) | 117 (15.7%) | 254 (18.8%) |
| Cumulative childhood trauma | 50 (14.9%) | 156 (20.9%) | 207 (15.3%) |

**Table S2.** Characteristics of the subsample of participants included in the analyses on symptom complexity according to recruitment strategy

| Demographics^a^ | Second strategy – Medical appointment  (n = 645) | Third strategy –  Online recruitment  (n = 1252) |
| --- | --- | --- |
| **Age**, mean (SD) | 29.6 (5.0) | 29.7 (4.0) |
| **Sex**, n (%) |  |  |
| Women | 545 (84.5%) | 1234 (98.6%) |
| Men | 100 (15.5%) | 18 (1.4%) |
| **Primiparous**, n (%) | 304 (47.4%) | 892 (71.3%) |
| **Marital status**, n (%) |  |  |
| In relationship | 612 (95.0%) | 1223 (98.1%) |
| Single | 32 (5.0%) | 24 (1.9%) |
| **Education level**, n (%) |  |  |
| No high school diploma | 29 (4.5%) | 10 (0.8%) |
| High school diploma | 54 (8.4%) | 57 (4.6%) |
| Collegial or professional training | 318 (49.5%) | 466 (37.3%) |
| University degree | 242 (37.6%) | 717 (57.4%) |
| **Ethnicity**, n (%) |  |  |
| White | 601 (94.8%) | 1210 (97.2%) |
| First Nations | 6 (0.9%) | 5 (0.4%) |
| Black | 9 (1.4%) | 5 (0.4%) |
| Hispanic | 12 (1.9%) | 12 (1.0%) |
| Asian | 1 (0.2%) | 4 (0.3%) |
| Other | 5 (0.8%) | 9 (0.7%) |
| **Annual household income**, n (%) |  |  |
| Can $34 999 or less | 88 (13.8%) | 73 (5.9%) |
| Can $35 000 – 64 999$ | 144 (22.5%) | 186 (14.9%) |
| Can $65 000$ - 85 999$ | 206 (32.2%) | 383 (30.7%) |
| Can $95 000 or more | 197 (30.8%) | 590 (47.3%) |
| **Prevalence of trauma**, n (%) |  |  |
| No trauma | 412 (63.9%) | 824 (65.8%) |
| One trauma | 104 (16.1%) | 236 (18.8%) |
| Cumulative childhood trauma | 129 (20.0%) | 192 (15.3%) |
| **Symptom complexity**, mean (SD) | -0.65 (3.02) | 0.32 (3.15) |
| **Psychiatric problems**, n (%) |  |  |
| No psychiatric problem | 544 (86.5%) | 1017 (81.2%) |
| One psychiatric problem | 61 (9.7%) | 143 (11.4%) |
| Comorbid psychiatric problems | 24 (3.8%) | 92 (7.3%) |

^a.^ Participants recruited through the first strategy were included in analyses on trauma prevalence only.
